# Enhancing Compliance With Preoperative Fasting Guidelines: A Closed-Loop Quality Improvement Initiative to Optimize Patient Safety and Outcomes

**DOI:** 10.7759/cureus.75250

**Published:** 2024-12-06

**Authors:** Abubakar I. Sidik, Alexander G Faybushevich, Md Limon Hossain, Em Samnang, Vladislav V Dontsov

**Affiliations:** 1 Cardiothoracic Surgery, Rossiskiy Universitet Druzhbi Narodov (RUDN University), Moscow, RUS; 2 Cardiology, I.M. Sechenov First Moscow State Medical University, Moscow, RUS; 3 Cardiovascular Medicine, Rossiskiy Universitet Druzhbi Narodov (RUDN University), Moscow, RUS; 4 Cardiothoracic Surgery, Moscow Regional Research and Clinical Institute, Moscow, RUS

**Keywords:** clear fluids, clincal audit, elective surgery, fasting times, preoperative fasting, quality improvement projects, solid food

## Abstract

Introduction

Preoperative fasting is essential in surgical care to reduce the risk of pulmonary aspiration during anesthesia. International guidelines, such as those from the American Society of Anesthesiologists (ASA) and the European Society of Anaesthesiology (ESA), recommend fasting durations of six hours for solids and two hours for clear liquids. However, adherence to these guidelines often varies in clinical practice, leading to prolonged fasting times that can negatively impact patient outcomes, including dehydration, hypoglycemia, discomfort, and delayed recovery. This quality improvement study aimed to evaluate adherence to international preoperative fasting guidelines within cardiovascular and thoracic surgery wards, identify barriers to compliance and implement targeted interventions to enhance adherence and improve patient outcomes.

Methods

The study was conducted using the Plan-Do-Study-Act cycle methodology. The study included 769 patients scheduled for elective procedures. A baseline audit of 90 (out of the 769) patients in September 2023 revealed that 86 (95.6%) and 82 (91.1%) fasted, respectively, from clear fluids for more than two hours and solid food for more than six to 12 hours, indicating significant non-adherence to the guidelines. This was followed by the implementation of an intervention plan that included staff training, patient education, and the introduction of a preoperative fasting checklist. Quarterly audits were conducted to assess the effectiveness of these interventions. Data on fasting durations, patient demographics, and associated complications were collected and analyzed using descriptive statistics and regression analysis.

Results

Following the intervention, there was a marked improvement in adherence, with significant reductions in prolonged fasting times across each quarter (p < 0.001). The last audit showed 529 (68.8%) fasted for more than two hours and 530 (68.9%) patients fasted for more than six to 12 hours, respectively, from clear fluids and solid food. Median fasting times were 4.1 hours for clear fluids and 12.0 hours for solid food. These improvements were accompanied by a reduction in postoperative complications such as dehydration, hypoglycemia, and postoperative nausea and vomiting. The study identified key barriers to adherence, including inadequate staff understanding of guidelines, inconsistent patient instructions, and changes in operating room schedules. The interventions effectively addressed these issues, though patients scheduled for afternoon surgeries continued to experience longer fasting durations than those scheduled for morning surgeries, suggesting the need for further adjustments to preoperative protocols.

Conclusion

This quality improvement study demonstrated that adherence to international preoperative fasting guidelines can be significantly enhanced through targeted interventions. The successful reduction in fasting durations and associated complications underscores the importance of continuous monitoring and protocol adjustments to align fasting practices with the best evidence, ultimately optimizing patient safety, comfort, and surgical outcomes.

## Introduction

Preoperative fasting is a standard practice in surgical care, designed to minimize the risk of pulmonary aspiration during anesthesia. For patients receiving regional anesthesia, they remain conscious after the procedure. However, if nausea and vomiting occur, there is a risk of aspiration. This risk is even higher in patients under general anesthesia. Therefore, preoperative fasting and fluid restrictions are necessary to mitigate this risk [1,2]. International guidelines, such as those provided by the American Society of Anesthesiologists (ASA) and the European Society of Anaesthesiology (ESA), recommend specific fasting times for solids and liquids to balance patient safety with comfort and well-being [3,4]. These guidelines typically suggest a fasting period of six hours for solid foods and two hours for clear liquids [3,4]. However, despite these clear recommendations, adherence to fasting protocols in clinical practice often varies, leading to prolonged fasting times that may adversely affect patient outcomes [5].

Prolonged fasting beyond the recommended guidelines can lead to a range of complications, including dehydration, hypoglycemia, increased patient discomfort, and a heightened stress response [6,7,8,9]. Additionally, extended fasting times have been associated with delayed recovery, increased postoperative nausea and vomiting (PONV), and impaired wound healing [10]. These complications not only compromise patient safety but also contribute to longer hospital stays and increased healthcare costs [10]. Prolonged fasting leads to hunger, thirst, and increased activity of the sympathetic nervous system, resulting in irritability and anxiety as reported by Napolitano et al. [11]. Prolonged fasting can also cause metabolic imbalances, as it reduces insulin secretion while increasing growth hormone and glucagon levels, disrupting glucose metabolism. Additionally, patients are prone to developing insulin resistance after surgery. Excessively long fasting can exacerbate this resistance, thereby reducing the body’s ability to fight infections and slowing tissue repair and wound healing [12].

The variability in adherence to fasting guidelines may be attributed to several factors, including inconsistent communication among healthcare providers, lack of standardized preoperative instructions, and variability in individual patient management [13]. Given the potential risks associated with prolonged fasting, there is a pressing need to assess current practices and implement strategies to improve adherence to evidence-based guidelines [14].

This quality improvement (QI) study aimed to evaluate the adherence to international preoperative fasting guidelines within surgical wards among patients scheduled for elective procedures, identify barriers to compliance, and implement targeted interventions to enhance adherence. By optimizing fasting practices, we seek to improve patient outcomes, reduce the incidence of fasting-related complications, and ensure that surgical care aligns with the best available evidence [3,4].

## Materials and methods

Study design

The study was conducted at A.A. Vishnevskiy Tretiy Sentralniy Voeniy Klinicheskiy Hospital, Russia. This study was designed as a QI initiative using the Plan-Do-Study-Act (PDSA) cycle methodology, which is a well-established framework in QI projects, enabling iterative testing of changes and continuous learning to improve processes [9].

Plan (Examine Current Approach, Identify and Develop Improvement Theory)

A baseline study was performed in September 2023 in three surgical departments to assess the extent to which international guidelines on preoperative fasting were followed. The baseline audit of 90 random patients hospitalized for elective procedures found poor adherence in all departments [15].

The intervention plan that was developed to tackle guidelines non-adherence included conducting workshops and training sessions for surgical ward staff, including nurses, anesthetists, and surgeons to reinforce the importance of adhering to fasting guidelines; introducing clear instructions for fasting times based on the scheduled surgery time; developing and distributing easy-to-understand educational materials for patients outlining the importance of fasting guidelines, including clear instructions on when to stop eating and drinking before surgery; introducing a preoperative fasting checklist to be used by nursing staff to confirm adherence to fasting guidelines during patient preparation for surgery.

Do (Test the Theory for Improvement)

The intervention plan was rolled out across all surgical wards, with adjustments made based on feedback and initial outcomes. If a patient is scheduled for surgery in the morning (AM patient), he/she is not allowed solid food after midnight but can have clear fluids until 06:30 am; if a patient is scheduled for surgery in the afternoon (PM patient), he/she can have solid food until 07:00 am and clear fluids until 11:00 am; information on fasting is conveyed to patients at pre-hospitalization and preoperative clinic visits; explanatory leaflets on fasting are also provided to patients.

After the implementation of the intervention plan, prospective follow-up studies were conducted monthly, and the results of every three consecutive months (October 2023 - June 2024) were analyzed together to evaluate improvement in adherence to international preoperative fasting guidelines. The primary outcome was the percentage of patients adhering to the recommended fasting times for solids (six to 12 hours) and clear liquids (two hours) as per international guidelines. The accepted fasting time from solid food in this study was six to 12 hours because the kitchens for the three departments operate from 8:00 AM to 7:00 PM, which made it difficult to strictly adhere to six hours. The secondary outcomes were incidence of complications associated with prolonged fasting, including dehydration, hypoglycemia, postoperative nausea and vomiting (PONV), and level of satisfaction with care received evaluated with a five-point Likert scale, ranging from “very dissatisfied” to “very satisfied”; patients scored their satisfaction level based on the degree of perioperative overall comfort, hunger, and thirst. 

Data collection

An initial survey of 36 nurses across three departments revealed that 29 of them (80.1%) instructed patients not to consume solid food after supper (7:00 PM) and to stop consuming clear fluids after 10:00 PM on the night before their surgical procedures. Patients were recruited preoperatively and were given a standardized questionnaire (supplementary information) as to obtain information on the form of fasting instructions they received (written only, verbal only, or both), the length of preoperative fasting, and complications of prolonged fasting times (Appendix 1). The total fasting duration (in hours) was measured from the start of fasting to the induction of anesthesia. The surgical approaches were minimally invasive, open, or conversion from a minimally invasive to an open technique. Patients’ demographics and hospitalization details were also collected.

According to the definition of surgical intervention by the National Confidential Enquiry into Patient Death and Outcome (NCEPOD), elective surgeries are those that are planned and scheduled prior to the patient's admission [16]. The inclusion criteria were adults (≥ 18 years) admitted for elective surgery, including those scheduled for same-day admission and overnight stays; the criteria for exclusion were individuals who were admitted to the theatre for acute care (NCEPOD “immediate, urgent, and expedited” classification), those who could not remember fasting time, patients undergoing redo surgeries during the same admission, patients with documented dementia, and pregnant and diabetic patients were excluded too.

Data analysis

Study (Use Data to Study the Result)

Data computation and analysis were carried out with Microsoft Excel 2021 (Microsoft Corporation, Redmond, Washington, United States) and IBM SPSS Statistics for Windows, Version 23 (Released 2015; IBM Corp., Armonk, New York, United States). Standard deviations (SD) and means were used to present data that were normally distributed. To assess statistical significance, the Mann-Whitney U test was employed. Non-parametric data were reported using medians and interquartile ranges (Q1, Q3). To evaluate the statistical significance between categorical variables, chi-squared analysis was applied, and p < 0.05 was considered statistically significant. The exponential of the regression coefficient (β) was computed to establish a ratio effect measure (eβ), which was subsequently used for calculating the effect measures. P-values in the multivariable analysis were determined using the likelihood ratio test. In subgroup analysis, procedures were categorized based on whether they were day cases or required overnight hospitalization. Morning surgeries (for AM patients) were scheduled between 8:30 am and 1:00 pm, while afternoon surgeries (for PM patients) were scheduled from 1:00 pm to 6:00 pm.

Ethics and permissions

As this study constituted a clinical audit of existing service provision, formal ethical approval was not necessary. However, it was registered with the hospital's local audit office, and it adhered to information governance and auditing standards. Before commencing the study, verbal consent was obtained from participants. While written consent was not acquired, participants were actively engaged in providing information regarding preoperative fasting.

## Results

Patients’ recruitment

**Figure 1:**
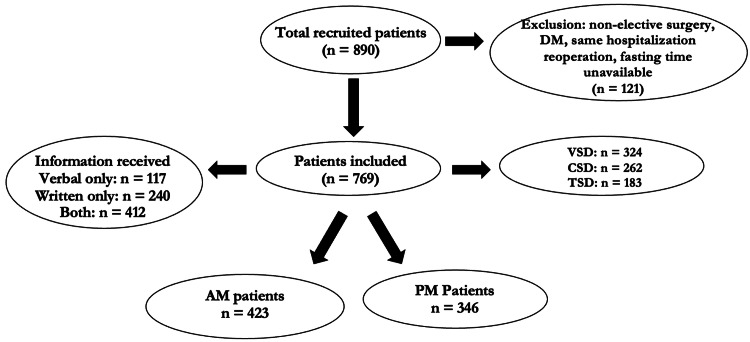
Participants’ recruitment. This figure demonstrates patients’ recruitment, inclusion and exclusion, division into the two groups and the form of instructions received. AM: patients with surgeries in the morning; CSD: cardiac surgery department; DM: diabetes mellitus; PM: patients with surgeries in the afternoon; TSD: thoracic surgery department; VSD: vascular surgery department

A total of 890 patients scheduled for elective operations were recruited into the study from September 2023 to June 2024; out of which 121 were excluded because they did not meet the inclusion criteria. Out of the remaining 769 patients (including 90 patients in the base audit) included in the study, 324 (42.1%) were from the Vascular Surgery Department (VSD), 262 (34.1%) from the Cardiac Surgery Department (CSD), and 183 (23.8%) from the Thoracic Surgery Department (TSD). Of the total, 412 (53.6%) received both verbal and written instructions on fasting, 117 (15.2%) confirmed they received only verbal instructions, and 240 (31.2%) received only written guidelines. Each patient received preoperative fasting instructions in one form or another (Figure 1).

Demographic profiles of patients

The majority of the patients scheduled for surgery were females (421 (54.7%)). More patients were scheduled for AM surgeries (423 (55.0%)) than PM surgeries (346 (45.0%)), with an average mean age of 56.6 ± 10.3 and 58.7 ± 9.7 years for AM and PM patients, respectively. In the majority of the cases, general anesthesia (521 (67.8%)) was used as opposed to 248 (32.2%) for regional anesthesia, as most cases (496 (64.5%)) were open procedures. Additionally, the mean body mass index of all patients was observed to be 27.2 (24.1, 30.3) kg/m². General anesthesia was used in 521 (67.8%) operations and regional anesthesia in some vascular procedures. (Table 1).

**Table 1:** Patients’ demographic data. This table is a representation of patients’ demographic features and the types of surgical procedures they received. AM: patients with surgeries in the morning; PM: patients with surgeries in the afternoon

Category	All Patients (n=769)	AM Patients (n=423, 55.0%)	PM Patients (n=346, 45.0%)
Age (years) - Mean ± SD	62.4 ± 10.3	59.2 ± 8.3	54.4 ± 6.7
Male - n (%)	348 (45.3)	191 (24.8)	157 (20.4)
Female - n (%)	421 (54.7)	232 (30.2)	189 (24.6)
Body mass index kg/m² - Median (Q1, Q3)	27.2 (24.1, 30.3)	25.6 (21.6, 28.3)	26.5 (21.1, 31.4)
Anesthetic type - n (%)			
General - n (%)	521 (67.8)	287 (37.3)	234 (30.4)
Local/regional - n (%)	248 (32.2)	136 (17.7)	112 (14.6)
Type of surgery - n (%)			
Vascular - n (%)	378 (49.1)	208 (27.0)	170 (22.1)
Cardiac - n (%)	243 (31.6)	133 (17.3)	110 (14.3)
Thoracic - n (%)	148 (19.3)	82 (10.7)	66 (8.6)
Surgical approach - n (%)			
Mini-invasive - n (%)	273 (35.5)	150 (19.5)	123 (16.0)
Open - n (%)	496 (64.5)	273 (35.5)	223 (29.0)

Analysis of preoperative fasting times of the entire cohort of patients

The overall median fasting times (Q1, Q3) were 4.1 (2.0, 5.6) hours for clear fluids and 12.0 (7.5, 14.7) hours for solid food. Significantly prolonged fasting, exceeding 12 hours, was observed in 72 patients (9.4%) for clear fluids and in 530 patients (68.9%) for solid food. Additionally, fasting periods longer than 24 hours for solid food were recorded in 76 patients (9.9%). Postoperative complications such as dehydration (evidenced by reduced urine output and hypotension), hypoglycemia, and postoperative nausea and vomiting (PONV), which was associated with prolonged fasting, and poor satisfaction with care received (a score <3 out of 5 points), showed improvement in each quarterly study (Table 2).

**Table 2:** Distribution of all patients according to months and duration of preoperative fasting. IQR is interquartile range (Q1, Q3). This table depicts the number of participants for each month of the audit and how long they fasted from clear fluids and solid food. PONV: postoperative nausea and vomiting; Sept: September 2023; Oct-Dec: October-December 2023; Jan-Mar: January-March 2024; Apr-Jun: April-June 2024

Fasting Times	Sept, n (%)	Oct-Dec, n (%)	Jan-Mar, n (%)	Apr-Jun, n (%)	Total, n (%)	Median (IQR)
Fasting times from clear fluids						
≤ 2 hours	4 (4.4)	61 (28.2)	83 (35.3)	92 (40.4)	240 (31.2)	-
2-4 hours	10 (11.1)	56 (25.9)	74 (31.5)	81 (35.5)	221 (28.7)	-
4-6 hours	35 (38.9)	47 (21.8)	40 (17.0)	32 (14.0)	154 (20.0)	-
6-12 hours	23 (25.6)	27 (12.5)	20 (8.5)	12 (5.3)	82 (10.7)	-
>12 hours	18 (20.0)	25 (11.6)	18 (7.7)	11 (4.8)	72 (9.4)	-
Total, n	90	216	235	228	769 (100.0)	4.1 (2.0, 5.6)
Fasting times from solid food						
6-12 hours	8 (8.8)	53 (24.5)	79 (33.6)	99 (43.4)	239 (31.1)	-
12-24 hours	67 (74.4)	138 (63.9)	136 (57.9)	113 (49.6)	454 (59.0)	-
> 24 hours	15 (16.7)	25 (11.6)	20 (8.5)	16 (7.0)	76 (9.9)	-
Total, n	90	216	235	228	769 (100)	12.0 (7.5, 14.7)
Postoperative complications						
Dehydration	20 (22.2)	27 (12.5)	24 (10.2)	15 (6.6)	86 (11.2)	-
Hypoglycemia	13 (14.4)	22 (10.2)	19 (8.1)	18 (7.9)	72 (9.4)	-
PONV	10 (11.1)	17 (7.9)	12 (5.1)	15 (6.6)	54 (7.0)	-
Satisfaction with care < 3	25 (27.8)	30 (13.9)	23 (9.8)	20 (8.8)	98 (12.7)	-

**Figure 2:**
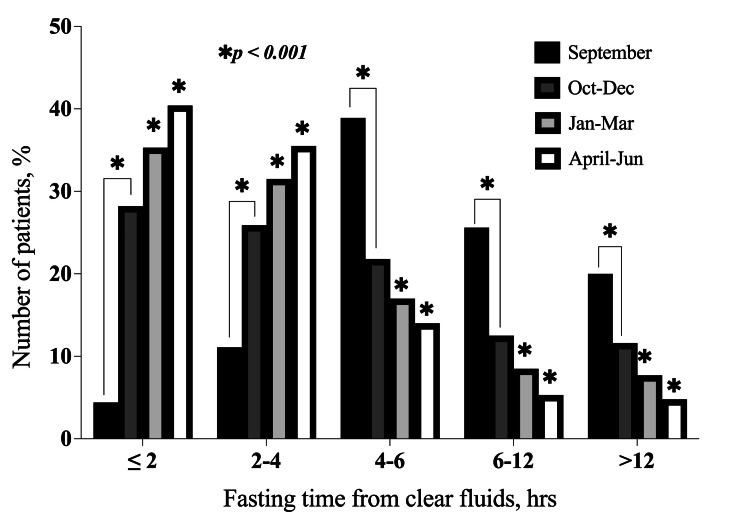
Distribution of fasting times from clear fluids according to months. This bar chart shows the changes in the number of patients that fasted from clear fluids for each of the time ranges in the subsequent months following the implementation of the action plan. *p < 0.001 for quarterly (Oct-Dec, Jan-Mar, and Apr-Jun, respectively) fasting times as compared to the baseline (September) audit. Sept: September 2023; Oct-Dec: October-December 2023; Jan-Mar: January-March 2024; Apr-Jun: April-June 2024

**Figure 3:**
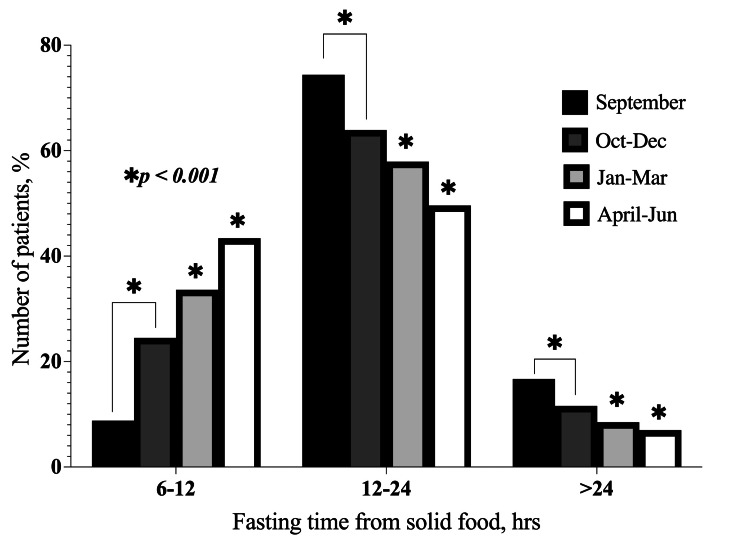
Distribution of fasting times from solid food according to months. This bar chart shows the changes in the number of patients that fasted from solid food for each of the time ranges in the subsequent months following the implementation of the action plan. *p < 0.001 for monthly (October, November, and December, respectively) fasting times as compared to the baseline (September) audit. Sept: September 2023; Oct-Dec: October-December 2023; Jan-Mar: January-March 2024; Apr-Jun: April-June 2024

Fasting times for both clear fluids and solid food were observed to decrease with each quarterly study, showing a significant improvement (p < 0.001) compared to the baseline in September. As a result, the number of patients who fasted for ≤ 2 hours from clear fluids and six to 12 hours from solid food increased after each quarterly study (Figure 2 and Figure 3). Additionally, there were no statistically significant differences in the fasting durations for solid food and clear fluids across the three departments (p = 0.091).

Among the 769 patients included in the study, 529 (68.8%) fasted from clear fluids for more than two hours, and 530 (68.9%) fasted from solid food for more than six hours. However, adherence to international fasting guidelines showed improvement with each quarterly study after the intervention plan was implemented. The reasons for non-adherence to fasting guidelines were identified as poor staff understanding and/or poor communication (among nurses, anesthetists, and patients) of the guidelines in 190 (25.1%) of 756 non-adherence cases, receiving only one form of instruction (either written or verbal) in 295 (39.1%) cases, reduced appetite due to preoperative stress and anxiety in 184 (24.4%) cases, and changes in the operating room schedule in 87 (11.5%) cases.

Comparison of fasting times between AM and PM patients

**Figure 4:**
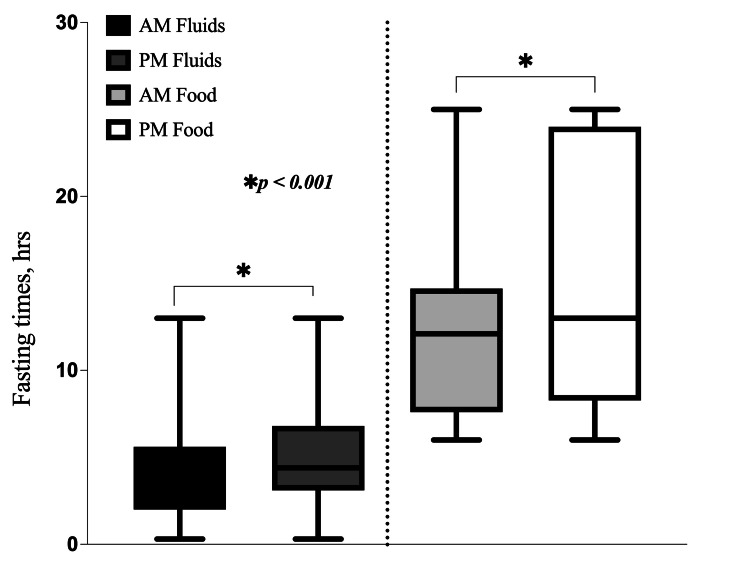
Comparison of preoperative fasting times from clear fluids and solid food in AM patients versus PM patients. This box and whisker plot represents a comparison of the length of fasting from clear fluids and solid food between AM and PM patients. *p < 0.001 for monthly fasting times from clear fluids and solid food between AM and PM patients. AM: patients with surgeries in the morning; PM: patients with surgeries in the afternoon

Regarding clear fluids, the majority of AM patients (149 (19.4%)) and PM patients (102 (13.3%)) fasted for four to six hours. For solid foods, most AM patients (212 (27.6%)) fasted for six to 12 hours, while most PM patients (136 (17.7%)) fasted for 12-24 hours. Overall, PM patients experienced significantly longer fasting times compared to AM patients. The median fasting times from clear fluids were notably higher in the PM group compared to the AM group: 4.4 (3.1, 6.8) hours versus 4.1 (2.0, 5.6) hours, respectively, with a p-value of < 0.001. Fasting times from solid food were also significantly longer in the PM group compared to the AM group: 13.0 (8.2, 24.0) hours versus 12.1 (7.5, 14.7) hours, respectively (Figure 4).

## Discussion

Findings

The results of this study highlight the importance of improving adherence to international preoperative fasting guidelines in cardiovascular and thoracic surgery wards similar to van Noort et al.'s report of their study [17]. Pre-intervention data revealed that a significant proportion of patients experienced fasting durations that exceeded recommended guidelines. Among the 769 patients studied, 68.8% fasted from fluids for more than two hours, and 68.9% fasted from solid food for more than six to 12 hours, which is above the recommended durations. The prolonged fasting times in this study contributed to complications such as dehydration, hypoglycemia, and postoperative nausea and vomiting (PONV). These complications are similar to those reported by Sisman et al., Buller et al., and Yeniay et al. [8,18,19]. These issues were particularly pronounced in PM patients, underscoring the need for more precise and individualized fasting protocols.

Several factors were identified as contributing to non-adherence to fasting guidelines, including poor staff understanding of the guidelines, insufficient or inconsistent patient instructions, stress and anxiety among patients, and changes in operating room schedules. These barriers highlight the complexity of maintaining adherence to fasting protocols in a dynamic clinical environment similar to those reported in a study by Robella et al. [20] and in other studies [21,22,23]. Previous studies have also reported that unpredictable surgery schedules, an exaggerated fear of aspiration, and unawareness of updated fasting guidelines among medical personnel are key reasons for the slow adoption of liberal fasting [24,25,26]. A national survey in Germany indicated that, despite awareness of the advantages of shorter fasting times, its implementation in anesthesia departments was poor [24]. Similarly, an Indian survey highlighted the lack of adoption of shorter fasting practices, despite anesthesiologists being aware of the updated guidelines [27]. Our findings are consistent with these previous studies. A national survey in Japan found that median fasting times were six to nine hours for clear liquids and 12-13 hours for solid food [28], which are similar to fasting times in this study.

The intervention, which included staff training, patient education, and the introduction of a preoperative fasting checklist, led to significant improvements in compliance. Over the three quarterly re-audits, fasting times were significantly reduced for both clear fluids and solid food (p < 0.001), with an increasing number of patients fasting for ≤ 2 hours for clear fluids and six to 12 hours for solid food, aligning more closely with international guidelines [3,4]. However, despite these improvements, the median fasting times of 4.1 hours for clear fluids and 12.0 hours for solid food indicate that some patients continued to exceed the recommended fasting durations. In contrast to the traditional fasting for solid food and clear fluids after midnight, shorter fasting protocols, allowing clear fluids up to two hours and solid foods up to six hours before surgery, have not been linked to risks such as regurgitation or aspiration [5].

Shorter fasting can significantly lower insulin resistance, prevent electrolyte imbalances, alleviate patient distress, and enhance comfort [9,29]; however, its widespread implementation in clinical practice remains limited. De Andrade Gagheggi Ravanini suggested that for patients undergoing surgery, a preoperative fasting period with a carbohydrate- and protein-enriched solution given two hours before surgery is both effective and safe, lowering insulin resistance without increasing the risk of aspiration [30]. Faria also confirmed that giving carbohydrate drinks two hours before surgery for patients is safe, helps reduce insulin resistance, and alleviates postoperative discomfort [31]. Additionally, Wang reported that administering preoperative oral carbohydrates rather than fasting improved thirst, hunger, and dry mouth symptoms in patients undergoing endoscopic procedures without increasing the risk of reflux [32].

Interestingly, no significant differences in fasting times were found across different departments (p = 0.091), suggesting that the intervention was uniformly effective across cardiac, thoracic, and vascular surgery wards. This finding highlights the broad applicability of the intervention, regardless of department-specific practices or patient demographics.

The study also showed that PM patients fasted significantly longer than AM patients. For clear fluids, the median fasting time was 4.4 hours for PM patients compared to 4.1 hours for AM patients. For solid food, PM patients fasted a median of 13.0 hours compared to 12.1 hours for AM patients, with the differences between these groups being statistically significant (p < 0.001). These findings, like those reported in other studies, suggest that patients undergoing afternoon surgeries are particularly vulnerable to prolonged fasting times, and additional adjustments to the fasting protocols for these patients may be necessary [8,33].

This reduction in prolonged fasting times contributed to a decrease in fasting-related complications, improving both patient outcomes and overall satisfaction with care. The use of the Plan-Do-Study-Act (PDSA) cycle allowed for continuous adjustments, demonstrating the effectiveness of quality improvement strategies in clinical practice [34].

However, despite these successes, some challenges persisted; PM patients continued to experience longer fasting durations than AM patients, primarily due to variations in operating room schedules and communication gaps regarding fasting instructions. Future interventions should focus on addressing these specific challenges, potentially by further tailoring fasting protocols based on surgery times and exploring the feasibility of staggered fasting instructions.

Additionally, one of the key barriers identified was inconsistent communication between healthcare providers and patients. Although improvements were seen, with the majority of patients receiving both verbal and written fasting instructions after the intervention, there remains room for refining how information is conveyed to ensure clarity and understanding. To manage preoperative fasting effectively, collaboration among anesthesiologists, nurses, and surgeons is essential [8]. A previous study reported that educating ward nurses and improving coordination among healthcare providers could significantly reduce unnecessary preoperative fasting in pediatric patients [35].

Act (Standardize the Improvement and Establish Future Plans)

Due to the effectiveness of the interventional plan in improving adherence to fasting guidelines, it was standardized and implemented as policy in the surgical wards. Recommendations that were made after the third study (in Apr-Jun 2024) included continuous quarterly study to evaluate adherence rates and presentation of outcomes to members of the departments, implementation of new measures such as the nursing team providing patients with a carbohydrate drink at least two hours prior to the surgery to shorten the fasting time, and establishment of better teamwork and communication between the surgical and theatre teams to minimize changes in the theatre lists at short notice in order to reduce fasting times. A graphical abstract that summarizes the findings of this study is provided (Appendix 2).

Limitations

This study had several limitations that must be considered when interpreting the results. First, the study was conducted within a single hospital setting, which may limit the generalizability of the findings to other institutions with different practices or patient populations. Second, the study primarily focused on elective surgeries, excluding emergency cases, diabetic patients, and pregnant patients, which may limit the applicability of the results to these groups. Third, while improvements were seen in fasting durations and complication rates, the study did not assess long-term outcomes such as the impact of improved fasting practices on overall recovery times, length of hospital stay, or healthcare costs. Lastly, the reliance on self-reported fasting times from patients could introduce recall bias, especially in cases where patients may not accurately remember their preoperative fasting periods.

## Conclusions

This study demonstrated that adherence to international preoperative fasting guidelines can be significantly improved through targeted interventions such as staff education, patient instructions, and the use of preoperative checklists. The observed reductions in fasting durations and associated complications, such as dehydration and PONV, underscore the importance of aligning clinical practice with evidence-based guidelines. However, the persistence of longer fasting times among afternoon surgery patients suggests that further protocol adjustments are necessary to ensure equitable adherence for all patients.

In conclusion, while this quality improvement initiative yielded positive results, continuous monitoring and iterative refinements are essential to maintaining these improvements. Addressing the specific needs of afternoon surgery patients and ensuring clear, consistent communication between staff and patients will be crucial for sustaining adherence to fasting guidelines and optimizing surgical outcomes.

## Figures and Tables

**Figure 5:**
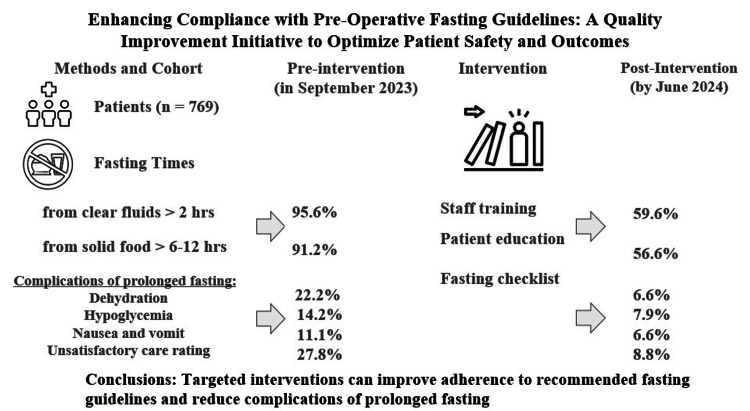
Graphical abstract.

## Appendices

Appendix 1 

**Table 3:** Supplementary material.

Please answer the following questions as best as you can remember	
QUESTION	ANSWER
To be completed by patient	
1. How many hours (or minutes) ago did you have your last drink?	
2. How many hours (or minutes) ago did you have your last meal (solid food)?	
3. How did you learn when to stop eating and drinking before your operation? Circle YES or NO.	
I saw it in my preoperative to-do list only	YES or NO
I was told at the preoperative clinic only	YES or NO
I saw it in my preoperative to-do list and was told at the preoperative clinic	YES or NO
To be completed by medical staff	
Postoperative complication due to prolonged fasting:	
Dehydration	YES or NO
Hypoglycemia	YES or NO
Postoperative nausea and vomiting	YES or NO
Reason for non-adherence to fasting time:	
Receiving only one form of instruction	YES or NO
Reduced appetite due to preoperative stress and anxiety	YES or NO
Changes in the operating room schedule	YES or NO
Other reason	

Appendix 2
